# Shedding Light on the Controversy Surrounding the Temporal Decline in Human Sperm Counts: A Systematic Review

**DOI:** 10.1155/2014/365691

**Published:** 2014-02-02

**Authors:** Marcello Cocuzza, Sandro C. Esteves

**Affiliations:** ^1^Department of Urology, University of São Paulo (USP), Rua Dr. Enéas de Carvalho Aguiar 255, 7o. Andar, Sala 710F, 05403-000 São Paulo, SP, Brazil; ^2^ANDROFERT, Andrology and Human Reproduction Clinic, Referral Center for Male Reproduction, Avenue Dr. Heitor Penteado 1464, 13075-460 Campinas, SP, Brazil

## Abstract

We systematically examined the evidence of declining sperm counts and the hypothesis that an increased exposure to environmental pollutants is responsible for such decline. Search engines, including PUBMED, MEDLINE, EMBASE, BIOSIS, and Cochrane library, were used to identify epidemiologic studies published from 1985 to 2013. We concluded that there is no enough evidence to confirm a worldwide decline in sperm counts. Also, there seems to be no scientific truth of a causative role for endocrine disruptors in the temporal decline of sperm production. Such assumptions are based on few meta-analyses and retrospective studies, while other well-conducted researches could not confirm these findings. We acknowledge that difficult-to-control confounding factors in the highly variable nature of semen, selection criteria, and comparability of populations from different time periods in secular-trend studies, the quality of laboratory methods for counting sperm, and apparently geographic variations in semen quality are the main issues that complicate the interpretation of the available evidence. Owing to the importance of this subject and the uncertainties still prevailing, there is a need not only for continuing monitoring of semen quality, reproductive hormones, and xenobiotics, but also for a better definition of fecundity.

## 1. Introduction

Carlsen and colleagues, in 1992, were the first to show robust evidence towards a semen quality decline. The aforementioned study opened this debate, and several independent studies have followed [1, 2]. In fact, more than one hundred articles have been published in the peer-reviewed literature in the past 50 years. Although many studies have reported a decline in sperm quality over time, others could not detect any changes [2]. The issue is still controversial since some studies were criticized for methodological errors, including bias in patient recruitment and in the methods of semen analysis [3]. As male fertility is to some extent correlated with sperm count, it would therefore be important to assess whether these findings were indeed reflecting a general reduction in male fertility [4].

Among fertile men, there are reports suggesting that the temporal decline in human sperm counts is independent of aging [5]. These changes have not been geographically homogeneously distributed, thus suggesting that specific factors, presented in some areas but not in others, might be related to a decline in the semen parameters [5]. Such factors include pollution, occupational exposure to industrial agents or heavy metals, and lifestyle risk factors including smoking, caffeine intake, and alcohol.

The biological significance of these temporal changes is emphasized by a concomitant increase in the incidence of genitourinary abnormalities such as testicular cancer, and also cryptorchidism and hypospadias, thus suggesting a growing impact of unknown factors with serious effects on male gonadal function [6]. In fact, malfunction of the male reproductive system could represent a high-quality sensitive marker of different hazards most likely to occur due to environmental rather than genetic factors. The human spermatozoa are the end result of sophisticated hormonal regulated biological processes. They are produced by a highly specialized cell line, initiated at puberty and continued throughout the man's entire life span in cycles. As such, the semen is a sensitive indicator of environmental, occupational, and lifestyle exposure that could exert direct toxic effects and hormonal disruption. Although damage might occur at any stage of life, early fetal life is a mainly critical time period, when the endocrine system is established and organs are developing [7].

We conducted this review to shed light on the controversy surrounding the temporal decline in the human sperm counts. We systematically examined the published evidence and critically analyzed the concept of “endocrine disruptor” as a cause of semen quality decline. For this, we performed an extensive search of studies examining the time-dependent change in human sperm counts using search engines and databases such as PUBMED, MEDLINE, EMBASE, BIOSIS, and Cochrane library. The overall strategy for study identification and data extraction will be based on the following key words: “epidemiology”, “male infertility,” “semen analysis,” “sperm count” “fertility” “xenobiotics,” “endocrine disruptors,” “humans,” “clinical studies”. Articles published in languages other than English were considered, but data that were solely published in conference or meeting proceedings, websites, or books were not included. Initial and final search dates were January 1985 and February 2013.

## 2. Epidemiological Temporal Trends in Sperm Counts

### 2.1. What Do Studies Supporting a Temporal Decline in the Sperm Counts Show?

As early as the 1980s, many health professionals raised concern over a possible deterioration of semen quality [8–11]. To better understand the dilemma, a meta-analysis of sixty-one articles including 14,947 men with no previous history of infertility was published by Carlsen and colleagues, in 1992. The authors concluded that the mean sperm count of healthy men had declined by 1% per year between 1938 and 1990 [1]. Furthermore, they reported a statistically significant decrease of a nearly 50% drop in sperm count from 113 × 10^6^/mL in 1940 to 66 × 10^6^/mL in 1990, and in the semen volume from 3.40 mL to 2.75 mL, using linear regression of data weighted by the number of men in each study. Their results indicated an even more pronounced decrease in sperm production than expressed by the decline in sperm density.

From 1995 and onwards, some investigators were skeptical about these findings, which prompted them to exam data in their own countries, mostly based on information coming from sperm banks or sperm donor registries [12, 13]. Their results were conflicting as some confirmed a trend towards a decrease in semen quality while others did not.

In 1997, Swan and colleagues published a reanalysis of global trend data [14]. The authors found significant decline in sperm density in the United States, Europe, and Australia after controlling for abstinence time, age, percentage of men with proven fertility, and specimen collection method. The sperm density decline in the United States (approximately 1.5% per year) and Europe/Australia (approximately 3% per year) was somewhat greater than the average reported by Carlsen and colleagues (approximately 1% per year). However, Swan and colleagues could not confirm a decline in non-Western countries, for which data were very limited. In 2000, an updated meta-analysis undertaken by the same authors confirmed the downward trend [15]. Swan and colleagues used similar methods to analyze an expanded set of studies as used by Carlsen and colleagues. Their new study included forty-seven English language reports published from 1934 to 1996, besides those reported in their previous study. They showed that the average decline in sperm count was virtually unchanged compared with Carlsen and colleagues' report and was analogous to their previous findings. The authors suggested that the reported trends were not dependent on the particular studies included by Carlsen and colleagues, and appended that the observed trends reported in the 1938–1990 period were also seen in the 1934–1996 period [1, 14, 15]. During the same period, there was a strong evidence for a worldwide increase in the incidence of testicular germ cell cancer, a disease linked to a decreased semen quality [6, 16, 17].

In Finland, a temporal decrease in semen quality was also observed in men from the general population in the period of 1998–2006. Men born more recently had decreased seminal parameters compared with the cohort born a few years earlier [18]. Their data were corroborated by other cross-sectional studies involving men of the general population in other European countries [19–38]. The reported declines of total count as well as sperm concentration varied from 16% to 31.5% during the study period (Table 1).

### 2.2. Temporal Sperm Count Decline: A Contrary Opinion

While several studies published prior to 1992, mainly involving cohorts of men seeking infertility in European countries, suggested a decline in the semen parameters [6–15], MaCleod and Wang, in the same period, reported on a large cohort of American men and showed contrary results [39]. These conflicting data remained a topic of debate over the years, albeit not achieving notable importance. It was after the report of Carlsen and colleagues [1], in 1992, that this debate gained worldwide media attention because of the magnitude of the findings showing a nearly 50% drop in sperm count from 1940 to 1990. Carlsen and colleagues suggested that the causes for the observed decline were related to exposure to compounds with estrogen-like activity and other environmental pollutants. Although part of the medical community reacted with skepticism and criticized their methods, their findings had a marked negative impact on the layman's imagination.

The main criticisms of the study by Carlsen and colleagues relied on the selection bias that might have occurred with the 61 assembled studies, so they would not have represented the underlying populations. The authors failed to include studies that showed no decline in the sperm counts, despite being available at the time of their meta-analysis. Surprisingly enough, the number of participants showing a decline in their semen parameters had been ten times larger than the counterpart showing no such decline. The authors also failed to account for geographic variation among the studies. Before 1970, all the studies came from the United States, with 80% of them from New York where sperm counts were the highest. After 1970, only three studies were from the United States. Many studies were from the third-world countries, where sperm counts were the lowest. Interestingly, a reanalysis of their meta-analysis accounting for this geographic variation could not confirm any decline in sperm counts [40]. Still, the application of an inappropriate statistical method involving linear regression might have contributed to their findings. If other mathematical models had been performed, different hypotheses would have been contemplated [41]. Carlsen and colleagues' data were robust for the last 20 years of the studied period only, in which all the models but the linear one suggested constant or slightly increased sperm counts [40]. Some have argued that, given the number of methodological flaws encountered, the study by Carlsen and colleagues should better be excluded from any review of data supporting a decline in sperm counts or other semen parameters. Yet, it was important to promote an incentive for researchers to explore the complex issue of semen quality.

Despite not being able to change the quality of data collected formerly, investigators could better deliberate studies to come. In 2012, Bahadur and colleagues published a study, initiated in 1996, which assessed the reproductive health of men from the general population [41]. In this large, prospective, and well-controlled study of semen quality of annual cohorts of young men from the general Danish population, a total of 4,867 individuals have been included. Surprisingly enough, statistically significant increases in the sperm concentration and total sperm counts over the past 15 years were detected. However, it was noted that men from the general population had significantly lower sperm concentrations, and also total sperm counts, than recently examined fertile men and men of a historical cohort of male partners of infertile couples. Still, only one in four men had optimal semen quality. Thus, the aforementioned authors concluded that there would be reason for concern about the future fertility of young Danish men, and their results seemed to be in line with growing needs of fertility treatment in Denmark [41].

The aforementioned Danish data, collected annually over a 15-year period, provided the best longitudinal semen data available yet. Since it comprised prospectively collected data from a well-defined male population that was examined according to a uniform protocol, it offers a much better basis for the evaluation of secular trends than retrospective data. In fact, the Danish study provided no indication that semen quality has changed during the past 15 years. However, it was of concern that men from the general population had significantly lower seminal parameters, in the new millennium, than recently examined fertile men as well as men of a historical cohort of infertile couples. Unfortunately, historical data on the semen quality of the general population of Danish men are lacking, except for the data collected by Hammen over 70 years ago [42].

When data from studies showing no decline in sperm counts were pooled [42–64], we noticed that twenty-four studies with a total number of 107,701 participants found no evidence of a decline in sperm counts (Table 2). In contrast, twenty studies with a total number of 79,884 reported an unambiguous decline in sperm count (Table 1). These numbers indicate that the studies reporting no decline, or even an increase in sperm counts, comprised approximately 30% more subjects than the studies reporting a decline in sperm counts published from 1995 onwards.

### 2.3. Cofounder Factors Influencing the Sperm Production in Humans

Studies of semen quality have been hampered by three sources of possible error. First, semen quality is highly variable. Attributes such as sperm concentration, semen volume, and sperm morphology vary widely not only between but also within individuals [65, 66]. Second, it is difficult to recruit men to volunteer for studies involving semen analysis; as such, selection biases are unavoidable. Studied populations have been selected from men who have provided semen samples for reasons such as donation to sperm banks, general population, evaluation for male factor infertility, and prevasectomy evaluation [41]. None of them represent a random sample of the population at large, and each presents a selection bias, although some are more likely to be biased than others [41, 67]. Third, studies rarely indicate whether seminal parameters have varied according to the geographic region [47, 68, 69]. The inability to include a truly random population represents an important source of potential methodological error [40]. At present, no data exist to elucidate the observed geographic variations in semen parameters.

Nevertheless, some studies have attempted to control for variables such as abstinence time, semen analysis, and collection methods [12, 13]. Longer abstinence periods lead to semen parameter changes, including a higher number of spermatozoa, semen volumes, and percentage of cells with abnormal morphology [19, 70, 71]. Intra- and interlaboratory variation in the methods of collection and analysis also exists [65, 72]. Semen analysis is a complex test which ideally should be carried out in Andrology laboratories. Minimum standards for laboratories performing semen analyses include the presence of experienced technicians, internal and external quality control, validation of test systems, and quality assurance during all testing processes. Accuracy, the degree whereto the measurement reflects the true value, as well as precision, the reproducibility of the results, is vitally important for clinicians who rely upon the values provided by the laboratory [73]. Intraindividual variation is a common feature and therefore at least two semen specimens should be included [65, 72–74]. Since sperm concentration is not normally distributed, proper transformations using logarithmic or cubic root would increase the power of the statistical analyses [74]. Moreover, confounding factors such as age, number of participants, and season of the year should ideally be taken into account to better compare data from different centers [12]. Interestingly, studies have shown seasonal fluctuations in sperm concentrations, with averages highest in springtime and lowest in the summer [56].

Another potential source of error is the inability to control for lifestyle factors, such as cigarette smoking or recreational drug use. An association between cigarette smoking and reduced seminal quality has been identified [75, 76]. Harmful substances including alkaloids, nitrosamines, nicotine, and hydroxycotinine are present in cigarettes, which results in the production of free radicals [77]. Increased levels of seminal oxidative stress have been observed in smokers compared with nonsmokers, possibly due to the significantly higher seminal leukocyte concentration in the former [78]. Higher numbers of sperm with DNA strand breaks have also been identified in smokers, which may be due to the presence of carcinogens and mutagens in cigarette smoke [79]. Chronic use of marijuana has also been associated with a trend toward elevated seminal fluid leukocytes [78]. Both experimental and human studies have demonstrated deleterious effects of tetrahydrocannabinol on sperm parameters including sperm concentration [80]. Regional or temporal trends in the use of tobacco and marijuana could have confounded the results of semen quality studies.

The aforementioned shortcomings justify the skepticism towards the semen quality studies published in the past 50 years. Most of them have not taken these potential sources of error into account, and, thus, their results lack credibility.

## 3. What Is the Likely Impact of Environmental Factors and Occupational Exposure on the Male Reproduction System?

During the past decades, the rapid growth of the chemical industry in both the developed and developing worlds has resulted in the release of a plethora of xenobiotics into the environment [7]. Xenobiotics are any alien molecules that are foreign to biological systems. Such substances, including pesticides, herbicides, cosmetics, preservatives, cleaning materials, private waste, and pharmaceutical products, have worked their way into our lives in a variety of forms. Even though consciousness of the biological risks of chemical toxicity has increased considerably in recent years, the majority of these chemicals have long half-lives and have been detected in the environment decades after their release.

Although the biological fallout of environmental pollution has centered on the risks of cancer, it has become evident that the reproductive system is also a major target. The disruption of germ cell differentiation seems to involve two fundamentally different routes of exposure. First, xenobiotics and other environmental factors such as radiation exert a direct effect on male germ cells within the mature testis. The extremely effective proofreading and DNA repair in stem cells means that the male germ line has one of the lowest spontaneous mutation rates in the body [81]. However, as these cells undergo meiosis their capacity for DNA repair is decreased, and their ability to respond to such damage by programmed cell death is gradually lost. As a consequence, once spermatozoa are released they can no longer rely on the protection previously afforded by the Sertoli cells. The male germ cell line is committed to spend a long journey of about 2 to 12 days in the epididymis and then up to 2 days swimming through the female reproductive tract searching for the oocyte. During this period, sperm are mainly vulnerable to DNA damage by a variety of environmental factors [81]. Hence, the spermatozoon is much more susceptible to damage than the oocyte, owing to its prolonged lonely existence and relative lack of protection, repair, and self-destructive mechanisms.

The second route by which xenobiotics exert their negative effects is by disrupting development of the reproductive tract of male fetuses. As a result, both germ cells and somatic tissues are affected, and a complex array of pathological changes collectively known as testicular dysgenesis syndrome (TDS) is observed in the male offspring. The features of TDS include poor semen quality, hypospadia, testicular cancer, and cryptorchidism. Although their exact mechanisms are not known, this syndrome is originated in the fetal life and its incidence seems to be increasing [82]. Some characteristics of TDS, such as low birth weight and retained placenta, have common risk factors, thus supporting the idea that they share the same pathophysiology mechanism involving perturbation of normal fetal development [83]. At present, little is known about the nature of xenobiotic-metabolizing enzymes in the male germ line as well as to which extent different groups of compounds induce genetic damage by oxidative stress mechanisms [7]. However, if xenobiotics were involved in causing testicular dysgenesis, they would act relatively early in fetal development.

Environmental estrogens, usually found in plants and in man-made products, competitively interact with the body's receptors for the natural estrogen. It is currently one of the most intensively researched compounds and source of the “estrogen hypothesis,” in which the apparent increase in the incidence of human male reproductive developmental disorders might have occurred due to increased estrogen exposure during the neonatal period [84]. It was hypothesized that environmental estrogens could suppress the production of follicle-stimulating hormone (FSH) by the fetal pituitary gland. As FSH stimulated the growth of Sertoli cells in the developing testes, the number of these cells would be consequently decreased [85]. Sertoli cells usually do not replicate, and each cell can only support the differentiation of a finite number of spermatozoa. Hence, a reduction in the size of this cell population could cause an irreversible impact on male germ cell development [84].

Environmental pollution, a major source of reactive oxygen species (ROS) production, has also been implicated in the pathogenesis of poor sperm quality [86]. In a study conducted by De Rosa and colleagues, tollgate workers with continuous environmental pollutant exposure had blood methaemoglobin and lead levels inversely correlated with sperm parameters in comparison to counterparts not exposed to comparable automobile pollution levels. These findings suggest that nitrogen oxide and lead, both present in automobile exhaust, adversely affect semen quality [87]. In addition, industrialization has resulted in deposition of highly toxic heavy metals into the atmosphere. Paternal exposure to heavy metals such as lead, arsenic, and mercury has been associated with decreased fertility and pregnancy delay [88, 89]. Oxidative stress was hypothesized to play an important role in the development and progression of adverse male reproductive health effects attributable to environmental exposure [90].

## 4. A Critical Appraisal on the Increased Temporal Incidence of Genitourinary Abnormalities 

Several reports indicate that an increase in the incidence of testicular cancer has occurred in the last 50 years, mainly in industrialized countries [17, 18, 91, 92]. Of note, European-descended populations seemed to be particularly affected, while East-Asian populations consistently have low rates of testicular cancer [6]. Interestingly, the incidence of female reproductive tract cancers, such as ovarian and uterine cancer, has remained constant while the incidence of cervical cancer has decreased during the same period of time [7]. Similar trends have also been observed in other developed countries [17]. Although no solitary hypothesis can be put forward to account for an unexpected increase in the incidence of testicular cancer, one of the possible explanations is the widespread use of ultrasound as a screening method in all fields of medical practice, including scrotal ultrasonography in urology [92, 93]. Another possible explanation could be the increased male longevity. However, testicular cancer is a disease of young men and is easily detected by self-examination.

A potential link between lower sperm counts and testicular cancer has arisen from the discrepancies in the incidence of male reproductive diseases between Danish and Finnish men [18]. Danish men seem to have the lowest sperm counts in Europe and also the highest incidence of testicular cancer and malformations of the genital tract such as hypospadias [94]. In contrast, the frequency of testicular cancer in Finnish men is practically three times lower. In addition, genital malformations are rare and the mean sperm counts are among the highest reported [95]. Currently, there is no convincing explanation why the reproductive fate of men in these two countries is so different, but a negative effect of environmental factors has been suggested [94]. Although the large variation in the incidence of testicular cancer among men of different racial and ethnic background suggests that genetic susceptibility is an important determinant [6], recent studies on smoking during pregnancy indicate strong geographical and temporal association between female smoking and testicular cancer in the offspring [96]. Such increases in testicular cancer in industrialized countries should alert urologists and andrologists to pay more attention to testicular symptoms, such as a painless testicular mass, testicular pain, swelling, hardness, or orchitis, and also to be more prone to recommend a testicular self-examination, particularly in adolescents and young adults [17].

## 5. Conclusions 

It has been suggested that sperm counts are declining over the last decades and that these changes might be responsible for a possible decline in fertility rates in the industrialized world. The reported decline in semen quality is a matter of great interest because it has been associated with a trend for an increased incidence of other male disorders, including testicular cancer and cryptorchidism. However, inter- and intraindividual biologic variability of sperm production, heterogeneity of the studied population, the paucity of information on subject characteristics, and the uncertainty in the quality and standardization of methods used for semen analyses make the interpretation of secular trends in semen quality extremely difficult as most studies have not taken these potential sources of error into account.

Occupational exposure to toxicants, including heavy metals, organic solvents, and pesticides, has been widely associated with reproductive dysfunction in males as well as in females. The possible mechanisms include both a direct effect on reproductive organs and an indirect effect resulting in hormonal imbalance that is crucial for growth, sexual development and many other essential physiological functions. Albeit environmental factors, whatever the route of exposure, can undoubtedly affect the male reproductive tract development and function, we must be circumspect of the wide range of behavioral, medical, and other factors that can potentially damage the male reproductive health. All these factors may contribute to a decrease in the fertility rates. To elucidate the causative effects of these observations, research efforts would require nontraditional collaboration between demographers, epidemiologists, clinicians, biologists, wildlife researchers, geneticists, and molecular biologists.

A critical appraisal of the studies included in our review indicated that it is unsound to assume that such trends are in place or that the observed effects might be linked to the lifestyle and environmental exposure to endocrine disrupters. We concluded that there is no enough evidence to confirm a worldwide decline in sperm counts or other semen parameters. Also, there is no scientific truth of a causative role for endocrine disruptors in the temporal decline of sperm production as observed in some studies. We conjecture that a definite conclusion would only be achieved if good quality collaborative long-term research was carried out, including aspects such as semen quality, reproductive hormones, and xenobiotics, as well as a strict definition of fecundity.

## Figures and Tables

**Table 1:** Summary of studies, published from 1995 up to date, showing an unambiguous decline in sperm counts.

Publication date	Author	Location	Study period	Number of participants
1995	Auger et al. [19]	France	1973–1992	1,351
1996	Irvine et al. [20]	Scotland	1984–1995	577
1996	Van Waeleghem et al. [21]	Belgium	1977–1995	416
1996	De Mouzon et al. [22]	France	1989–1994	7,714
1996	Menchini-Fabris et al. [23]	Italy	1970–1990	4,518
1996	Adamopoulos et al. [24]	Greece	1977–1993	2,385
1999	Ulstein et al. [25]	Norway	1975–1994	4,072
1998	Bonde et al. [26]	Denmark	1986–1995	1,196
1999	Bilotta et al. [27]	Italy	1981–1995	1,068
1999	Zhang et al. [28]	China	1983–1996	9,292
2003	Almagor et al. [29]	Israel	1990–2000	2,638
2003	Vicari et al. [30]	Italy	1982–1999	716
2005	Lackner et al. [31]	Austria	1986–2003	7,780
2007	Sripada et al. [32]	Scotland	1994–2005	4,832
2008	Liang et al. [33]	China	1980–2005	5,834
2009	Feki et al. [34]	Tunisia	1996–2007	2,940
2010	Molina et al. [35]	Argentina	1995–2004	9,168
2012	Geoffroy-Siraudin et al. [36]	France	1988–2007	10,932
2012	Haimov-Kochman et al. [37]	Israel	1995–2009	2,182
2013	Mendiola et al. [38]	Spain	2001–2011	273

**Table 2:** Summary of studies, published from 1995 up to date, not showing a decline in sperm counts.

Publication date	Author	Location	Study period	Number of participants	Sperm count*
1996	Bujan et al. [44]	France	1977–1992	302	NC
1996	Vierula et al. [45]	Finland	1967–1994	5,719	NC
1996	Paulsen et al. [46]	United States	1972–1993	510	NC
1996	Fisch et al. [47]	United States	1970–1994	1,283	↑
1997	Berling and Wölner-Hanssen [48]	Sweden	1985–1995	718	↑
1997	Benshushan et al. [49]	Israel	1980–1995	188	NC
1997	Handelsman [50]	Australia	1980–1995	689	NC
1997	Rasmussen et al. [51]	Denmark	1950–1970	1,055	NC
1997	Zheng et al. [52]	Denmark	1968–1992	8,608	NC
1998	Emanuel et al. [53]	United States	1971–1994	374	NC
1998	Younglai et al. [54]	Canada	1984–1996	48,968	NC
1999	Andolz et al. [55]	Spain	1960–1996	20,411	NC
1999	Gyllenborg et al. [56]	Denmark	1977–1995	1,927	↑
1999	Zorn et al. [57]	Norway	1975–1994	1,108	NC
1999	Acacio et al. [58]	Slovenia	1983–1996	2,343	NC
2000	Itoh et al. [59]	United States	1951–1997	1,347	NC
2001	Costello et al. [60]	Japan	1975–1998	711	NC
2002	Marimuthu et al. [61]	Australia	1983–2001	448	NC
2003	Chen et al. [62]	India	1990–2000	1,176	NC
2003	Carlsen et al. [63]	United States	1989–2000	551	↑
2005	Mukhopadhyay et al. [64]	Denmark	1996–2001	158	NC
2010	Esteves et al. [65]	India	1980–2000	3,729	NC
2011	Axelsson et al. [43]	Sweden	2000–2010	511	NC
2012	Jensen et al. [42]	Denmark	1996–2010	4,867	↑

*↑: significant increase; NC: no significant change.
